# The clinical significance of sCD14-ST for blood biomarker in neonatal hematosepsis: A diagnostic accuracy study

**DOI:** 10.1097/MD.0000000000006823

**Published:** 2017-05-05

**Authors:** Ting Xiao, Li-Ping Chen, Li-hua Zhang, Fu-Huang Lai, Li Zhang, Qun-feng Qiu, Rong-Liang Que, SiSi Xie, Ding-Chang Wu

**Affiliations:** a Department of Clinical Laboratory; b Department of Neonatal Unit, Fujian Longyan First Hospital, Longyan First Affiliated Hospital of Fujian Medical University, Longyan, Fujian, China.

**Keywords:** gram-negative bacteria, gram-positive bacteria, hematosepsis, neonate, sCD14-ST

## Abstract

Hematosepsis is a systemic inflammatory response syndrome (SIRS) with suspected or confirmed infection, which is the most common infectious disease in clinical neonatal intensive care unit. As the rapid development of neonatal hematosepsis caused by various basic diseases, the mortality rate is high, and there are some sequelae.

We report the lasted study to date with 96 cases from Fujian Longyan First Hospital between 2013 and 2015. The aim of our study is to explore the value of soluble cluster of differentiation 14 subtype (sCD14-ST) in whole blood for differential diagnosis of neonatal hematosepsis at an early stage, and used in evaluation of the severity about sepsis combined with acute physiology and chronic health evaluation II (APACHE-II) score, procalcitonin (PCT), C reactive protein (CRP), and leukocyte (WBC).

In our cohort, all cases met the diagnostic criteria for hematosepsis specific for newborns. We selected 42 neonates with hematosepsis, 54 neonates with nonhematosepsis, 44 noninfectious SIRS neonates, and 53 healthy neonatal controls. Which were determined the sCD14-ST, PCT, CRP, and WBC of all samples before treatment. Then assign the APACHE-II score for the all samples before and after treatment.

The study shows, sCD14-ST levels were significantly higher in hematosepsis than nonhematosepsis group (*t* = −2.112, *P* = .041). Meanwhile, sCD14-ST levels were significantly higher in neonatal hematosepsis than in noninfectious SIRS group and controls (*χ*^2^ = 57.812, 68.944, *P* < .01). However, sCD14-ST in hematosepsis group was positively correlated with APACHE-II score (*R*-value = 0.415, *P* < .01). During treatment, the sCD14-ST level was decreased obviously along with APACHE-II score, PCT, CRP, and WBC (*χ*^2^ = 35.019, 78.399, 52.363, 25.912, 7.252, all *P* values <.01). The area under the curve (AUC) of sCD14-ST was 0.942. The differences in ROC^AUC^ of sCD14-ST compared with PCT, CRP, and WBC were statistically significant (*Z* = −6.034, −4.474, −5.722, all *P* values <.01). The sensitivity and specificity of sCD14-ST were 95.2% and 84.9%, respectively.

sCD14-ST could be a blood biomarker for early identification and disease valuation in newborns hematosepsis infection; and its diagnostic value is superior to other laboratory indexes.

## 1 Introduction

Systemic inflammatory response syndrome (SIRS) triggered by infectious causes will be resuited in hematosepsis.^[1]^ The source of infection can be placental abruption at birth, mother-to-fetus transmission, living environment, hospital infection, etc. A number of pathogenic bacteria may invade blood circulation, then release and disseminate toxins by reproduction. It will be terrible if we only treat with amount of broad-spectrum antibiotic as normal infection.^[2]^

Hematosepsis will appear and develop rapidly, and may result in systemic multiple organ failure; the mortality of hematosepsis is extremely high and about 30% to 50% neonatal deaths annually.^[3]^ In premature, hematosepsis could affect various system growth seriously and cause various abdominal organs disease.^[4,5]^ With the improvement of medical technology, the researchers have found more than one hundred laboratory markers applied to clinic.^[6]^ Although, blood culture is still the “gold standard” for diagnosis of hematosepsis, which can be used to identify the type of bacteria in a timely and accurate manner, providing a reliable basis for rational clinical practice of antibacterial drugs. However, such identification takes much time and is susceptible to contamination and antibiotic. The laboratory indexes of hematosepsis have been researched for many years.

As a marker rarely reported, soluble cluster of differentiation 14 subtype (sCD14-ST) is one of the main receptors of lipopolysaccharide (LPS) and the fragment of leukocyte differentiation antigen CD14, including soluble CD14 (sCD14) and membrane CD14 (mCD14).^[12,15]^ Application of sCD14-ST in judgment of severity and prognosis of neonatal hematosepsis is still absent. In this study, the type of bacteria was identified through the detection of sCD14-ST level in whole blood of the neonate with hematosepsis; meanwhile, comparison of area under curve (AUC) was made among sCD14-ST, procalcitonin (PCT), leukocyte (WBC), and C reactive protein (CRP), respectively; the value of sCD14-ST for diagnosis and severity assessment of neonatal hematosepsis at an early stage was evaluated.

## 2 Patients and methods

### 2.1 Our case series

The neonates visited the pediatric intensive care unit (PICU) of Fujian Longyan First Hospital, China, from August 2013 and March 2015. Data were collected by a single author in 96 neonates with infectious SIRS (including 42 hematosepsis, and 54 nonhematosepsis), there were 50 males (52.1%) and 46 females (47.9%), whose gestational age was 37.9 ± 2.55 weeks on average. These neonates included 79 full-term infants and 17 premature infants (28 weeks < gestational age < 37 weeks), with mean birth weight of 3008.33 ± 596.64 g. Cases with congenital diseases or recently treated with immunosuppressant, and treated for more than 3 days in other hospitals were excluded. All cases in the study met the diagnostic criteria for hematosepsis specific for children (including newborns) established by international pediatric experts based on the physiological features of children at different ages in 2005.^[7]^ Fifty-three health controls included 28 males (28/53, 52.83%) and 25 females (25/53, 47.16%), with average gestational age of 38.77 ± 1.41 weeks and birth weight of 3238.25 ± 545.72 g. Cases with mal-development or congenital diseases were excluded. Details are shown in Table 1.

**Table 1:**
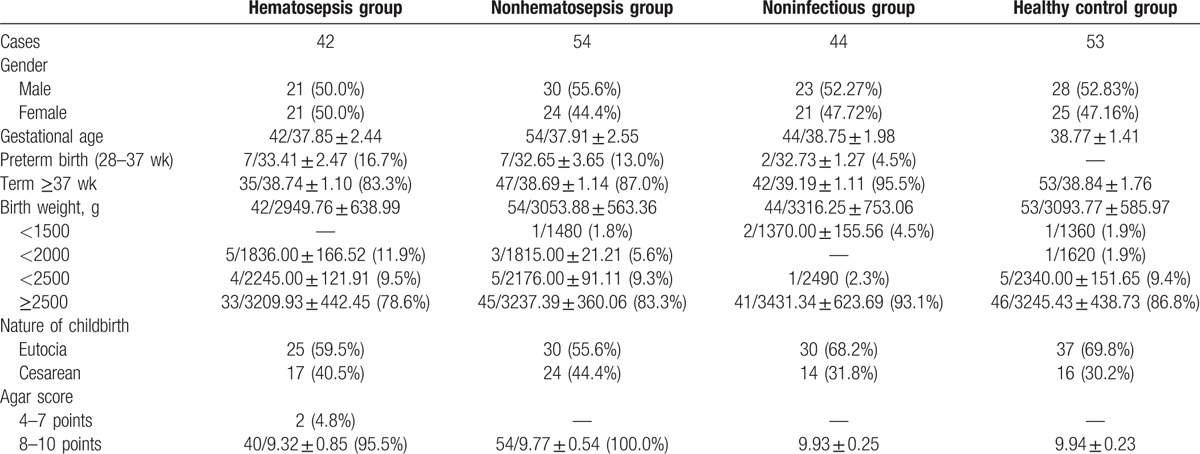
Basic data composition of neonates.

### 2.2 Methods

#### 2.2.1 Sample collection

All subjects were collected of 2 mL venous blood in EDTA anticoagulant tubes and 5 mL venous blood in separation gel tubes.

#### 2.2.2 Reagents and instruments

sCD14-ST, PCT, CRP, and WBC were determined by PATHFAST Immunoanalyzer and Reagents (fluorescent enzyme immunoassay, Mitsubishi Chemical Medience Corporation, Tokyo, Japan), Immunochramato Reader HR 201 (Immuno-chromatographic assay, B.R.A.H.M.S. GmbH, Germany), Olympus AU2700 Automated Chemistry Analyzer (Immune scatter turbidimetry, Beckman Coulter, Inc., Osaka, Japan), and Sysmex XE-5000 Automated Hematology Analyzer (Electrical impedance method and optical method, SYSMEX Corporation, Kobe, Japan), respectively. BD BACTEC FX Instrument and BD Phoenix-100 (New York) were used for blood culture and bacteria identification. Blood culture bottles and bacteria identification panels were purchased from Becton, Dickinson and Company (New York).

Collection and processing of clinical data: The name, gender, age, medical history, and blood biochemical test results of patients were recorded. Acute physiology and chronic health evaluation II (APACHE-II) scores were obtained for hematosepsis group.^[8,9]^ Blood specimens were taken from neonates with hematosepsis and then cultured in bacteriology room. True or false-positive results were determined in combination with clinical symptoms. This study was approved by the Ethics Committee of Longyan First Affiliated Hospital of Fujian Medical University, Longyan, China. Written informed parental consent was obtained for all patients.

### 2.3 Statistical analysis

SPSS 19.0 statistical software was used, and normality test was done for all data. Measurement data were expressed in median (quartile) [M (*P*_25_–*P*_75_)] or mean ± SD ( ± *s*). Kruskal–Wallis *H* test was used for 3-group comparison, and paired *t* test was used for pairwise comparison. Receiver operating characteristic curve (ROC) analysis was performed on data,^[10]^ and the AUC of 1.0 suggests optimal test index, AUC of a diagnostic test without any significance is 0.5. In general, a diagnostic test is of low value when its ROC-AUC is between 0.5 and 0.7, of medium value when its ROC-AUC is between 0.7 and 0.9, and of great value when its ROC-AUC is higher than 0.9. *P* < .01 indicated significant statistical difference. Pearson test was used for correlation analysis.

## 3 Results

### 3.1 Basic data of neonates (Table 1)

**Figure 1:**
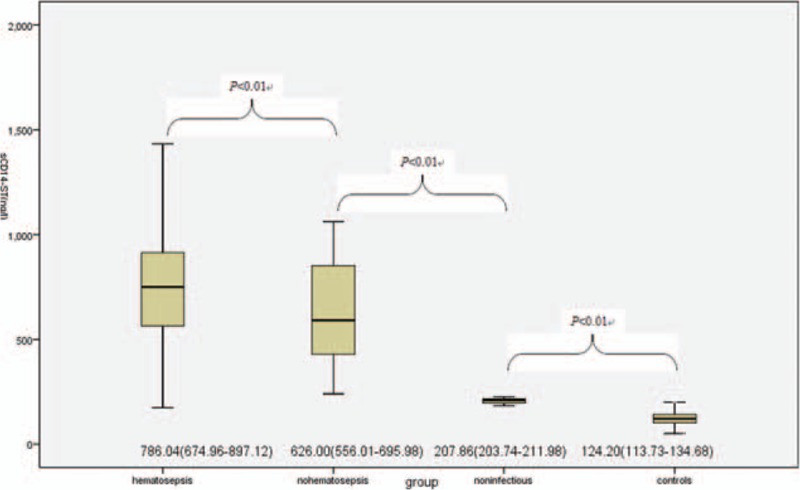
Comparison of sCD14-ST levels (ng/L) among hematosepsis group and nonhematosepsis group, noninfectious group and healthy control group.

Significant differences in sCD14-ST levels were observed among hematosepsis group and nonhematosepsis group, noninfectious group and healthy control group (all *P* values < .01), seen in Fig. 1.

Comparison of all laboratory indexes before treatment between hematosepsis and nonhematosepsis group showed that there was significant difference in sCD14-ST levels of 2 groups (*P* < .05), but not in PCT, CRP, and WBC (*P* > .05), seen in Table 2.

**Table 2:**
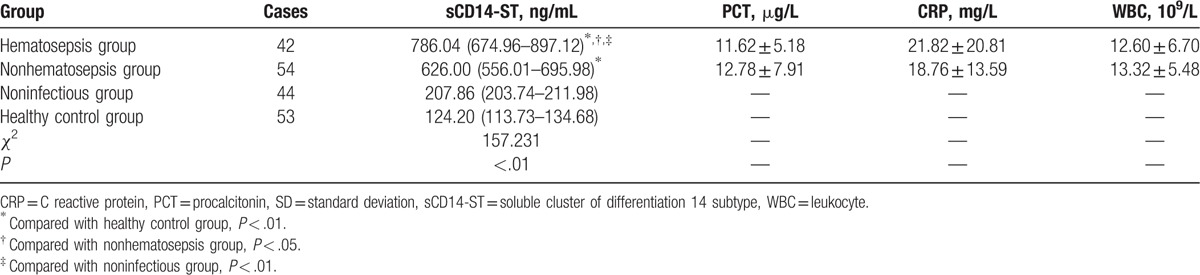
Comparison of sCD14-ST, PCT, CRP, and WBC between hematosepsis group and nonhematosepsis group (quartiles, mean, SD).

**Figure 2:**
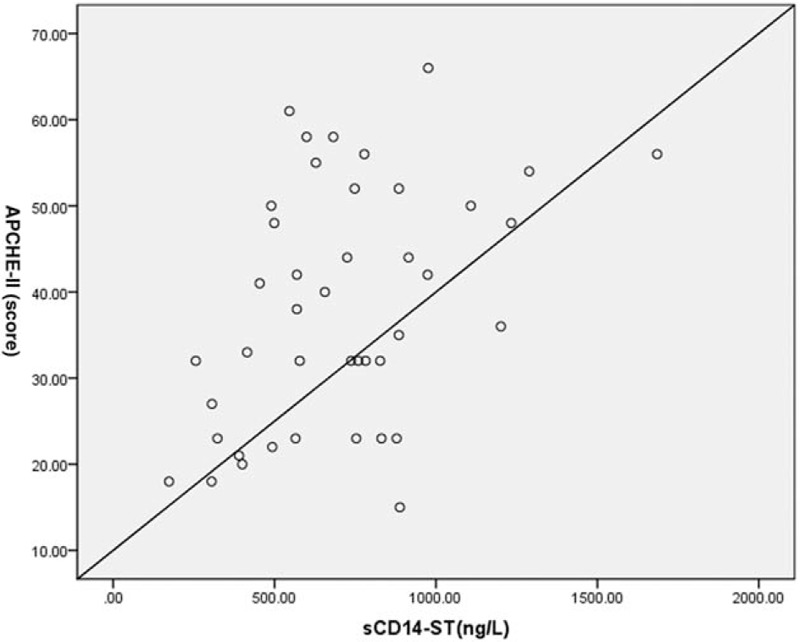
Correlation analysis of sCD14-ST with APACHE-II score in hematosepsis group.

The correlation of sCD14-ST with APACHE-II score obtained from neonates in hematosepsis group before treatment showed that sCD14-ST had significantly positive correlation with APACHE-II score in hematosepsis group (*R* = 0.415, *P* < .01), seen in Fig. 2.

**Figure 3:**
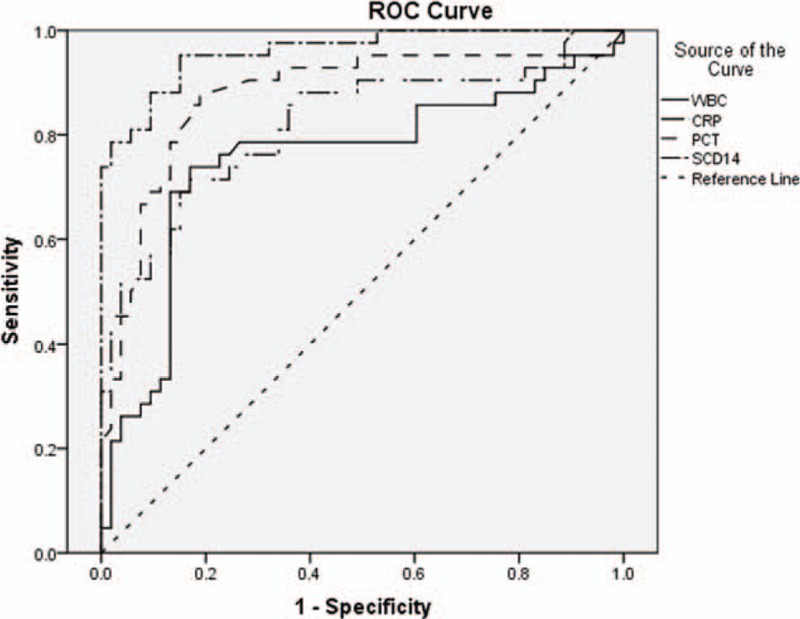
ROC curves of sCD14-ST, WBC, CRP, and PCT results in 42 neonates with hematosepsis.

ROC curve analysis of sCD14-ST, WBC, CRP, and PCT for 42 neonates with hematosepsis ROC curves are shown in Fig. 3. Evaluation of diagnostic capabilities of sCD14-ST, WBC, CRP, and PCT is seen in Table 3. Differences in AUC^ROC^, sensitivity and specificity among 4 indexes were statistically significant (*P* < .01).

**Table 3:**
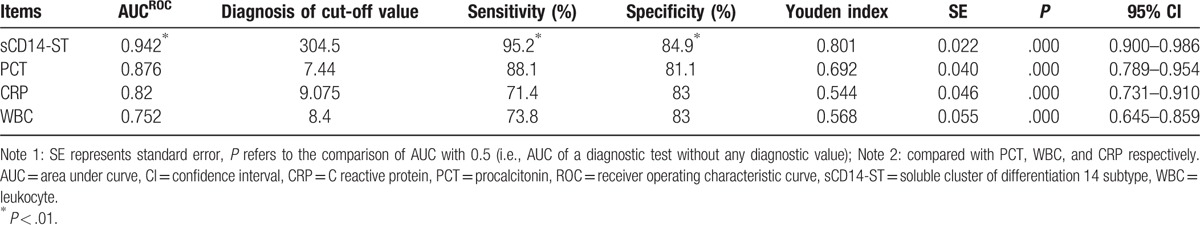
ROC curve analysis and evaluation of diagnostic capabilities of 4 indexes in 42 neonates with hematosepsis.

Results showed that sCD14-ST, PCT, WBC, CRP, and APACHE-II score obtained from 42 neonates with hematosepsis were all apparently decreased before and after treatment (*P* < .01), seen in Table 4.

**Table 4:**
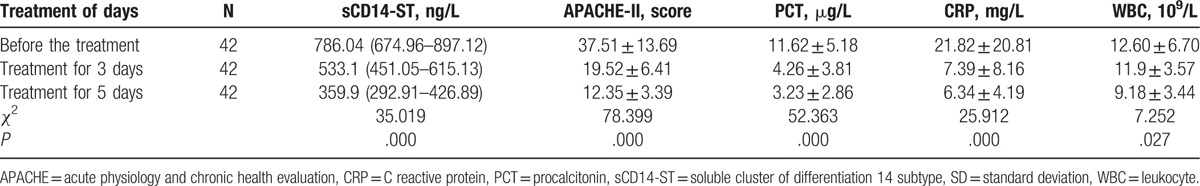
Changes in sCD14-ST, PCT, WBC, CRP, and APACHE-II score of neonates with hematosepsis before and after treatment (quartiles, mean, SD).

In 42 neonates with hematosepsis, sCD14-ST in gram-positive bacteria and gram-negative bacteria group were both higher than those in healthy control group (*P* < .01), but sCD14-ST between gram-positive bacteria and gram-negative bacteria group was not statistically different (*P* > .05), results seen in Table 5.

**Table 5:**
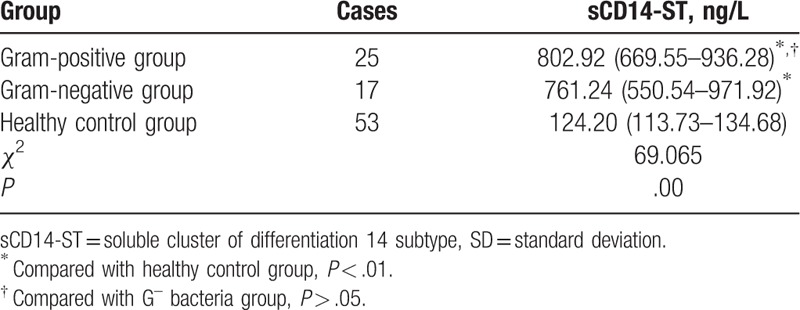
Comparison of sCD14-ST between G^+^ bacteria group and G^–^ bacteria group in 42 neonates with hematosepsis (quartiles, mean, SD).

After blood culture in 42 cases of neonatal hematosepsis, gram-positive cocci were found to be in the majority, accounting for 60% (25/42), followed by gram-negative bacilli, accounting for 40% (17/42), results seen in Table 6. And there is 17 cases of neonatal hematosepsis were premature infant, the bacteria were mainly gram-negative bacilli, accounting for 59% (10/17), followed by gram-positive cocci, accounting for 41% (7/17), results seen in Table 7.

**Table 6:**
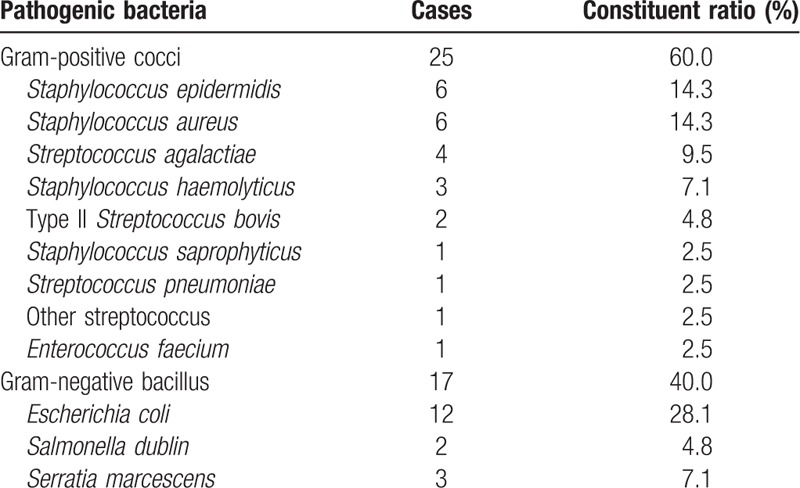
Distribution and constituent ratio of pathogenic bacteria in 42 neonates with hematosepsis (n = 42).

**Table 7:**
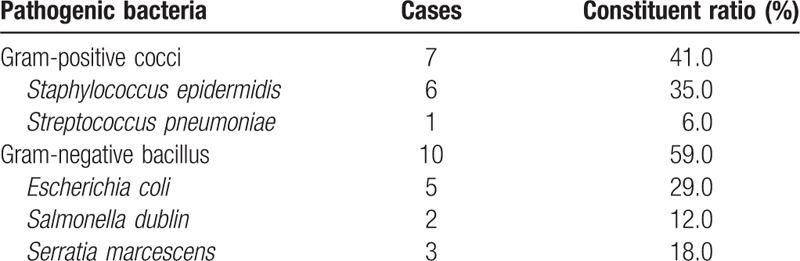
Distribution and constituent ratio of pathogenic bacteria in premature infants of hematosepsis group (n = 17).

## 4 Discussion

With diverse and nontypical manifestations in clinic, hematosepsis caused by suspicious or confirmed infectious SIRS in a special group on neonatal period, which imposes a serious threat to the lives of neonates. Therefore, it is urgent to find out a laboratory blood marker for fast and accurate diagnosis.^[11]^

As one of indexes of systemic inflammatory response, sCD14-ST level in whole blood plays an important role in the process of bacterial infection, particularly, it is closely related to the progress of hematosepsis associated with systemic multiple organ failure.^[13]^ Our study shows that the sCD14-ST level in whole blood in hematosepsis group was obviously higher than those in nonhematosepsis group, noninfectious SIRS group, and healthy control group (*P* < .01) suggesting that it could be an indicator for identifying hematosepsis and nonhematosepsis.

Resch et al conformed that PCT level is the most sensitive index for diagnosis of hematosepsis within 12 hours after birth and may be used for diagnosis of hematosepsis at the early stage. However, the diagnosis result based on fair increase of PCT level in the premature infant may be false negative.^[14]^ The significant difference in sCD14-ST level (*P* < .05) and no difference in the levels of PCT, CRP, and WBC (*P* > .05) were found in our study before treatment between hematosepsis and nonhematosepsis group. It appeared that sCD14-ST has guiding significance to some extent in identifying hematosepsis from nonhematosepsis. Due to lack of anaerobic and fungal culture conditions, we collected the samples with such bacteria into nonhematosepsis group by mistake. Thus, the reliability of data should be verified by the culture of various bacteria and the research on a large quantity of samples collected from the neonates at different ages.

With the ROC curve analysis compared with sCD14-ST, PCT, CRP, and WBC. We found that the value of in diagnosis of sepsis, sCD14-ST AUC is the largest, meanwhile the sensitivity is 95.2% and the specificity is 84.9%. It suggested that sCD14-ST has certain advantages in early and effective diagnosis of neonatal sepsis.

As one of the main receptors of LPS, sCD14-ST combines with the LPS in bacterial cell wall, particularly in SIRS caused by gram-negative bacteria infection.^[12]^ Although blood culture has been recognized as the “gold standard” for diagnosis of hematosepsis, false positive or negative result may appear due to the effect of various external factors.^[16,17]^ Some scholars have found that sCD14-ST level could help to identify the neonatal hematosepsis caused by gram-positive bacterial infection or gram-negative bacterial infection.^[18]^ However, our study showed that there was no statistically significant difference in sCD14-ST levels between gram-positive bacterial group and gram-negative bacterial group in 42 cases of neonatal hematosepsis (*P* > .05). Such inconsistency may be attributed to less samples, species specificity, hospital infection, etc. Therefore, we need to test a large quantity of samples to confirmed whether sCD14-ST level in whole blood can be used to identify the hematosepsis caused by gram-positive or gram-negative bacteria infection.

However, it has confirmed that sCD14-ST as a blood marker can be used for the research and judgment of severity of neonatal hematosepsis and the prognosis thereof.^[19–21]^ Currently, sCD14-ST level has been reported as an index for diagnosis and evaluation of hematosepsis in the infant with a low birth weight.^[22]^ As there are only a few infants with a low birth weight among the subjects of this study, the above-mentioned report is not supported and further research on a large quantity of samples collected from infants with different birth weights is yet to be conducted.

In our study, APACHE-II score was assigned to each neonate in hematosepsis group before treatment, and the correlation between APACHE-II score and sCD14-ST level was analyzed. The results showed that in hematosepsis group, sCD14-ST was positively correlated with APACHE-II score (*R*-value = 0.415, *P* < .01) and the sCD14-ST level, APACHE-II score, PCT, WBC, and CRP before and after treatment were decreased obviously (*P* < .01), which is consistent with the findings that sCD14-ST level in the patient with hematosepsis markedly increased and such increase was correlated with the severity and prognosis of hematosepsis.^[23]^ According to these findings, sCD14-ST level can be taken as an important laboratory index for early diagnosis of infectious diseases and evaluated the severity of neonatal hematosepsis with sCD14-ST level in combination with APACHE-II score.

Additionally, the constituent ratio of bacterial in 42 cases of neonatal hematosepsis demonstrated that gram-positive cocci comprised 60% (25/42) of infections, followed by gram-negative bacilli, accounting for 40% (17/42). The results showed that the neonate with hematosepsis was mainly infected with gram-positive bacteria (*Staphylococcus*), among which *Staphylococcus aureus* and *Streptococcus agalactiae* were clinically significant and other staphylococcocci such as *Staphylococcus epidermidis* and *Staphylococcus saprophyticus* were considered as contaminants, and then with gram-negative bacteria (predominantly *Escherichia coli*). The results are consistent with the findings of Srinivasa and Arunkumar that *S aureus* as gram-positive bacteria were the main factor leading to neonatal hematosepsis,^[24]^ and with the reports that different pathogenic bacteria of neonatal hematosepsis vary in distribution.^[20,21]^ The research of Stoll et al^[13]^ mentioned that approximately 73% of patients infected with streptococcus were term infants, which is almost consistent with the result of this study. However, we found no streptococcus other than *S agalactiae*, which may be related to species difference or the limited quantity of samples. We found the bacteria in the premature infant with hematosepsis were mainly gram-negative bacteria (bacilli), accounting for 59% (10/17), followed by gram-positive bacteria (cocci), accounting for 41% (7/17), which is consistent with the findings of the scholars that the hematosepsis in the premature infant was mainly caused by gram-negative bacteria infection^[25,26]^; and such infection was dominated by *E coli*, which is also consistent with the description of Stoll et al^[13]^ that about 81% of patients infected with *E coli* were premature infants. Since premature infants are more susceptible to hematosepsis due to immature tissues and organs, low birth weight, staying in ICU for a long period of time, blue-light irradiation, subcutaneous catheter indwelling, central venous catheterization and mechanical ventilation, the blood culture result of premature infants is considered to be related to hospital infection.^[27,28]^

Not all of bacteria caused hematosepsis can be detected by traditional blood culture because of maternal's own factor and the difference in microbial species.^[29]^ Biedenbach et al^[30]^ found that neonatal hematosepsis caused by *Klebsiella pneumoniae* and *Pseudomonas aeruginosa* was attributed to overuse of antibiotic. But our study did not find any of the above 2 bacteria, which may be related to the living environment and hospital infection of mother and child; meanwhile, considering limited number of samples studied, the research and analysis of a large quantity of samples is yet to be done.

## 5 Conclusion

Compared with the conventional laboratory indexes, the determination of sCD14-ST in whole blood can be used for early diagnosis of hematosepsis and nonhematosepsis in neonates. It is worthy of clinical application and promotion in monitoring the therapeutic effect of hematosepsis and evaluating the severity with APACHE-II.
